# dCaP: detecting differential binding events in multiple conditions and proteins

**DOI:** 10.1186/1471-2164-15-S9-S12

**Published:** 2014-12-08

**Authors:** Kuan-Bei Chen, Ross Hardison, Yu Zhang

**Affiliations:** 1 Center for Comparative Genomics and Bioinformatics, The Pennsylvania State University, University Park, PA 16802, USA; 2 Department of Computer Science and Engineering, The Pennsylvania State University, University Park, PA 16802, USA; 3 Departments of Biochemistry and Molecular Biology, Pennsylvania State University, University Park, Pennsylvania 16802, USA; 4 Department of Statistics, The Pennsylvania State University, 422A Thomas, University Park, PA 16802, USA

## Abstract

**Background:**

Current ChIP-seq studies are interested in comparing multiple epigenetic profiles across several cell types and tissues simultaneously for studying constitutive and differential regulation. Simultaneous analysis of multiple epigenetic features in many samples can gain substantial power and specificity than analyzing individual features and/or samples separately. Yet there are currently few tools can perform joint inference of constitutive and differential regulation in multi-feature-multi-condition contexts with statistical testing. Existing tools either test regulatory variation for one factor in multiple samples at a time, or for multiple factors in one or two samples. Many of them only identify binary rather than quantitative variation, which are sensitive to threshold choices.

**Results:**

We propose a novel and powerful method called dCaP for simultaneously detecting constitutive and differential regulation of multiple epigenetic factors in multiple samples. Using simulation, we demonstrate the superior power of dCaP compared to existing methods. We then apply dCaP to two datasets from human and mouse ENCODE projects to demonstrate its utility. We show in the human dataset that the cell-type specific regulatory loci detected by dCaP are significantly enriched near genes with cell-type specific functions and disease relevance. We further show in the mouse dataset that dCaP captures genomic regions showing significant signal variations for TAL1 occupancy between two mouse erythroid cell lines. The novel TAL1 occupancy loci detected only by dCaP are highly enriched with GATA1 occupancy and differential gene expression, while those detected only by other methods are not.

**Conclusions:**

Here, we developed a novel approach to utilize the cooperative property of proteins to detect differential binding given multivariate ChIP-seq samples to provide better power, aiming for complementing existing approaches and providing new insights in the method development in this field.

## Background

A central problem in molecular biology is to understand how proteins and DNA interact to regulate genes that lead to phenotypic diversity. Given massively parallel second generation sequencing technologies [1-3], huge collection of data are being generated for a wide diversity of regulatory elements genome-wide (such as transcription factors, epigenetic marks, genetic variants, DNaseI hypersensitivity, and transcriptomes). The huge datasets enable us to comprehensively study the mechanics of gene regulation among multiple factors across many cell types and experimental conditions simultaneously [4,5]. Key regulators functioning in specific cells/conditions and their impacts to gene expression can therefore be pinpointed with substantially improved power and specificity than conventional approaches [6,7]. New hypotheses about the dynamics of the epigenetic landscape during differentiation can consequently be derived and tested to illuminate previously intractable issues in the genetics of disease susceptibility [7-9].

How to effectively analyze the huge collection of epigenetic data sets is a major challenge. Many computational methods have been developed to make binary presence-absence calls for detecting protein-DNA interaction events in a single condition [10-13], which are then used in down-stream analysis to study gene regulation and differentiation [14,15]. Although the binary calls of interaction events, such as transcription factor (TFs) binding, have been shown to aid the study in gene regulation, such an approach is problematic because the same event may be called differently due to the choice of thresholds and the data scale in different samples. Alternatively, quantitative methods (e.g. ANOVA) [16] have been used to study differential or condition-specific binding variations. Such an approach is often overly simplistic that does not account for the possible heterogeneity in the real data. When there are few replication data available, it is also difficult to obtain good estimates of the heterogeneous variance of the noise, which then greatly reduces the sensitivity of the study.

More recent approaches have been developed to quantify binding variations among multiple features or samples [16-20]. Kasowski et al. used pairwise ANOVA to study the genome-wide binding strengths of POL2 and NFkappaB between each pair of the 10 individuals at merged binding regions [16]. In addition to using traditional statistical tests, some other studies adopted methods developed previously to identify differential expressed genes to detect differential bindings in ChIP-seq experiments [21]. For example, a gene stability measure M was defined by Vandesompele et al. as the average pairwise standard deviation between any particular gene and all other genes [22]. Ozer et al. adapted the stability measure to determine most variable regions binding regions across all 50 ChIP-seq experiments. However, this stability measure does not provide a measure for statistical significance and it's also difficult to interpret the value M across different experiments. Taslim et al. developed DIME that takes normalized differences of ChIP-seq counts in each genomic bin as input and used a finite mixture model (non-differential, negative/positive differential) to identify genes with differential binding for a specific protein in two conditions [18,19]. Instead of using genomic bins in Taslim et al. for normalization, Shao et al. [23] later proposed a method called MAnorm to improve data normalization in ChIP-enriched regions (peaks) based on the assumption that most common peaks have the same binding strength to build the rescaling model similar to DIME. They used a Bayesian model developed for detecting differential gene expression to determine the significance of differential binding [24]. While DIME and MAnorm have been developed for comparing binding signals for a single protein, a more recent method dPCA [25] uses a small number of principle components to summarize the pattern of difference in multiple proteins between two samples.

These methods, however, are limited to two-sample comparisons. As the number of conditions increases, pairwise comparison will become computationally expensive. Combining and interpreting the data from pairwise results can also be inconsistent. To overcome the limitation of pairwise comparison, DBChIP was recently developed to allow comparison in multiple conditions [17]. The method first clusters nearby predicted binding sites into consensus sites across multiple conditions, and then uses a generalized linear model with Negative Binominal distribution to detect differential TF binding across samples. DBChIP however only considers one protein at a time. Biologically, protein-DNA interactions are correlated among regulators. Combined patterns of signals for different epigenetic features can provide more insights towards the regulatory mechanisms in cells. A most recent method, jMOSAiC [20], jointly analyzes multiple epigenetic features in multiple samples. jMOSAiC internally partitions signals into presence or absence events, and hence only detects qualitative variation.

**Figure 1:**
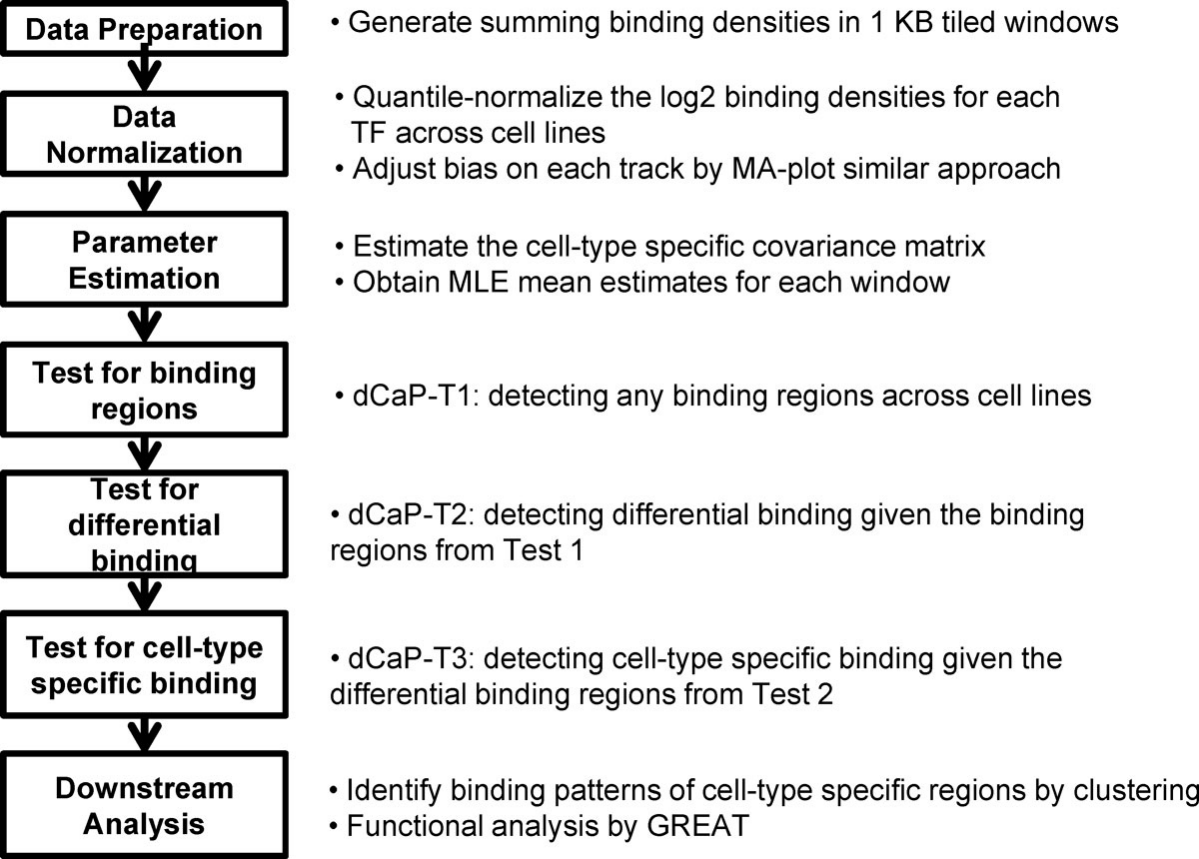
**Flow chart of dCaP**.

In need of a systematic, powerful and flexible method for simultaneous integration and comparison of ChIP-seq data for multiple epigenetic features in multiple conditions, we introduce a unified method, dCaP (Differentiation among Conditions and Proteins), to detect and quantify protein-DNA interaction events and their variations by treating multiple tracks of features as multivariate samples sequenced in multiple conditions. Here, a "condition" can be an experimental condition, or more broadly, a tissue, a cell type, or a sample with phenotypes. dCaP uses locally weighted smoothing to estimate the heterogeneous variance of noises in the data, such that it is free from the unrealistic constant variance assumption, but at the same time it retains power even for small sample experiments. dCaP can work with or without replicates. After normalization, dCaP utilizes a three-step log-likelihood ratio test (Figure 1) to jointly identify multiple protein-DNA interaction events in multiple conditions genome-wide, detect regions with significant signal variations in at least one feature across conditions, and classify the detected regulatory loci for condition-specificity. While existing methods detect condition-specific events via binary calls, dCaP detects quantitative variations.

We applied dCaP to two real studies to demonstrate the power and the utility of dCaP. The first data set consists of 30 data tracks (of ChIP-seq and DNase-seq) from the human ENCODE consortium. We chose five human cell lines: GM12878, HUVEC, HeLa-S3, HepG2, K562; and three regulatory factors in each cell line: transcription factor CTCF, RNA polymerase II (POL2) and DNaseI sensitivity of the chromatin (DHS) [5]. Each factor in each cell type has 2 replicates. By applying dCaP, we identified 194,840 statistically significant occupancy regions genome-wide, among which 33,205 contained differential signals and 16,452 were further cell-type specific. Clustering analysis revealed two major clusters of occupancy patterns in the five cell lines. One cluster showed consistently enriched signals for all three factors and they were enriched near genes showing functions highly relevant to each of the cell types. The other cluster showed depleted signals for all three factors and they were strongly associated with homozygous deletions in the three cancer cell lines (HeLa-S3, HepG2, K562). The results by dCaP indicated potential regulatory regions responsible for cell-type specific biological behaviours. We further applied dCaP to detect differential TAL1 occupancy in response to GATA1 restoration in two mouse erythroid cell lines. Conventional approaches use peak callers (6) to identify binary binding events in each cell line separately and then take the difference between cell lines. Such solutions are sensitive to the peak calling thresholds and cannot detect quantitative variation in TAL1 occupancy. Using dCaP, we identified a large set of novel differential TAL1 occupancy missed by the binary peak calling method, and they were highly significantly associated with GATA1 occupancy as well as differential gene expression. dCAP is simple but powerful. It can be widely applied to many studies for simultaneous comparison of multivariate ChIP-seq data across multiple conditions.

## Methods

### Datasets

*Human ENCODE Data: *Mappings of short reads of CTCF, POL2 and DNaseI to the human genome (hg18) were collected from the ENCODE open chromatin data from the Duke/UNC/UT-Austin/EBI ENCODE group for 5 selected cell lines [5]. The five chosen cell lines are: K562 erythroleukemia cells [26], HeLa-S3 cervical carcinoma cells [27], HepG2 hepatoblastoma cells [28], GM12878, a B-lymphoblastoid cell line (http://1000genomes.org) and primary (non-transformed) human umbilical vein endothelial cells (HUVEC) [29]. Among the 5 cell lines, the first three are cancer cell lines and the last two are relatively normal cell lines. We used two replicates in each of the 15 data types (5 cell lines × 3 factors), resulting in 30 tracks of data in total.

*Mouse ENOCDE Data: *we obtained read counts by the ChIP-seq experiments of TAL1 in two mouse erythroid cell lines (G1E, G1E-ER4) from Wu et al. [15,30]. Each cell line contains two data replicates. The union of TAL1 binding peaks reported from Wu et al. [15] in the two conditions were used to detect the differential binding by dCaP. The cell line G1E is derived from in vitro differentiated *Gata1*-null mouse ES cells, proliferates as committed erythroid progenitors and undergoes terminal differentiation upon restoration of *Gata1 *expression [15] and its subline G1E-ER4 expresses an estrogen-activated *Gata1*-estrogen receptor (ER) transgene.

### Data pre-processing

*Human ENCODE Data: *We used F-Seq [31] to generate continuous tag sequence density estimation in 1-bp resolution for all 30 data tracks. We then computed the sum of tag sequence density in 1-kb tiled windows across the human genome for hypothesis testing of common and differential occupancy across cell lines. Blacklist regions from UCSC assembly gaps and the Duke excluded regions were found to have the anomalous high signals and thus excluded in our analysis. This resulted in 2,821,390 windows for testing across the human genome, excluding the Y chromosome. We further took log_2_-transformed density to reduce the skewness of the data. A small constant (0.1) was added to the value when taking the log_2 _to avoid numerical problems. Based on the log_2 _density values in 1-kb windows, we further performed quantile-normalization [32] on the 10 data tracks (5 cell lines, 2 replicates) for each factor to make datasets across cell types comparable.

The quantile normalization does not necessarily remove the systematic bias within replicates. We further applied LOESS (locally weighted regression) normalization [33] on the log_2 _transformed density of each data track. Let $x_{j,k,r}^{\left(i\right)}$ denote the data value in window *i*, cell line *j*, factor *k *and replicate *r*, and let ${\bar{x}}_{k}^{\left(i\right)}$ denote the mean of factor *k *across all cell lines and replicates in window *i*. We applied LOESS function by regressing $\left(x_{j,k,r}^{\left(i\right)}-{\bar{x}}_{k}^{\left(i\right)}\right)$ on the factor mean ${\bar{x}}_{k}^{\left(i\right)}$, for data in all windows in each data track. The LOESS estimate ${\tilde{x}}_{j,k,r}^{\left(i\right)}$ was then subtracted from $x_{j,k,r}^{\left(i\right)}$so that the new values (at the same level of ${\bar{x}}_{k}^{\left(i\right)}$) has mean 0 across windows. For large datasets, LOESS computation can be slow, and the fitted curve can be overly smooth or curvy at different values using a fixed smoothness parameter. To increase computing speed and to fit local curves better, we partitioned the data values into bins and we subsampled data with equal frequency from each bin. This prevented the fitting curve being biased by the unbalanced data where the data points were much denser at low signals and sparser at high signals. We sampled 1% of the total data using 25 bins and the maximum number of data points per bin was bounded by the total number of sampled points divided by the number of bins. For the LOESS parameters, we used span = 0.5 with locally linear fit (degree =1).

*Mouse ENOCDE Data: *we obtained the processed data (the read counts) from Wu et al. (6). We took the average log2 value of the read counts within each detected binding region and performed quantile-normalization. The values were then used as the input to dCaP for testing differential binding.

### Method notations

We propose three log likelihood ratio tests (LRT) under asymptotic multivariate normality assumption. For notation consistency, we use lowercase/uppercase bold letters to denote vectors and matrices, respectively, and we use uppercase regular letters for constants. Although Negative Binomial distributions or other distributions with over-dispersion parameters are often used for modeling *-seq data, they cannot be easily adopted to model the joint distribution of multivariate signals with covariance structures. In our case, covariance is abundant among related proteins, and multivariate normal distributions can conveniently account for the covariance among signals. Multivariate normality is also reasonable when testing the means of samples over many conditions, and when proper data transformation is used on the *-seq data, such as log-transformation, averaging signals over intervals, or quantile transformation to z-scores.

Let $X^{\left(i\right)}=\left(x_{1,1}^{\left(i\right)},\;x_{1,2}^{\left(i\right)},\ldots ,\;x_{C,P-1}^{\left(i\right)},\;x_{C,P}^{\left(i\right)}\right)$ denote a *C *by *P *data matrix containing log_2_-transformed signals after proper normalization in *C *conditions with *P *variables, for condition *j *= 1 ... *C*, variable *p *= 1 ... *P*, and windows *i *= 1 ... *N *across the genome (*C*≥2, *P*≥1). For each condition *j *in window *i*, the column vector $x_{j}^{\left(i\right)}$ consists of *K *factors with *R *replicates (*K*≥1, *R*≥1), and therefore $x_{j}^{\left(i\right)}$ has length P = K*R and is represented as $x_{j}^{\left(i\right)}=\left(x_{j,1,1,}^{\left(i\right)}\;x_{j,1,2}^{\left(i\right)},\ldots ,\;x_{j,K,R-1}^{\left(i\right)},\;x_{j,K,R}^{\left(i\right)}\right)=\left(x_{j,1}^{\left(i\right)}\;x_{j,2,}^{\left(i\right)}\ldots ,x_{j,P}^{\left(i\right)}\right)$. We assume that each vector $x_{j}^{\left(i\right)}$ follows a *P*-dim multivariate normal distribution, $x_{j}^{\left(i\right)}\sim N\left({û}_{j}^{\left(i\right)},{\hat{\sum }}_{j}^{\left(i\right)}\right)$ with a condition specific mean ${û}_{j}^{\left(i\right)}$ and a covariance matrix ${\hat{\sum }}_{j}^{\left(i\right)}$ in the *ith *window, for *j *= 1 ... *C*. The mean vector ${û}_{j}^{\left(i\right)}$ has a restriction that the means of replicates of the same factors are identical. We first estimate ${û}_{j}^{\left(i\right)},{\hat{\sum }}_{j}^{\left(i\right)}$ along with ${û}_{0}^{\left(i\right)}$ under the null hypotheses. We then use log-likelihood ratio tests to detect potential common and differential regulatory binding events across the genome. Hereafter, we use "binding" to denote a protein-DNA interaction event, even though our method is technically applicable to detect any signal enrichment and variation in general.

### Estimation of covariance matrix

To obtain the covariance matrix for condition *j *in window *i*, we first estimate a condition specific (but not window specific) correlation matrix ${\hat{R}}_{j}$, which is a *P *by *P *matrix that captures potential relationships between different factors and replicates. Let $X_{j}$ denote the *N *by *P *data matrix in condition *j *in all genomic windows. We want to compute ${\hat{R}}_{j}=Cor\left(X_{j}^{bk}\right)$, where $X_{j}^{bk}$ denotes a data matrix from genomic background (e.g., no binding) and *Cor(.) *denotes correlation. Since $X_{j}$ contains a mixture of binding and non-binding data and binding regions are unknown, estimating background $X_{j}^{bk}$ from $X_{j}$ is not straightforward. Directly estimating ${\hat{R}}_{j}$ by $Cor\left(X_{j}\right)$ will lead to bias, because the binding signals can greatly inflate the correlation between factors/replicates.

Assume that $X_{j}=X_{j}^{bk}+m$, where *m *denotes an unknown vector of the means of binding signals independent of genomic background. We have

(1) $$Cov\left(X_{j}\right)=Cov\left(X_{j}^{bk}\right)+Cov\left(m\right)$$

We estimate *m *by ${\bar{X}}_{j}$, which is an *N *by *P *matrix containing the mean of each factor across conditions (the mean is identical within replicates for each TF). As a result, we obtain

(2) $$\hat{Cov}\left(X_{j}^{bk}\right)=Cov\left(X_{j}\right)-Cov\left({\bar{X}}_{j}\right)$$

and then we compute ${\hat{R}}_{j}$ by standardizing $\hat{Cov}\left(X_{j}^{bk}\right)$. On the other hand, if input controls are available, ${\hat{R}}_{j}$ can be obtained directly from the controls. When pre-defined binding regions are available for directly testing differential binding, $X_{j}$ became a *N *by *P *data matrix containing the binding signals in all binding regions in condition *j*. To obtain ${\hat{R}}_{j}$ in this case, we first subtract mean of each factor across conditions in each row, then we take the bottom 50% of rows ranked by the total variance of centred data to calculate the correlation matrix.

Given ${\hat{R}}_{j}$, we compute the window-specific covariance matrix, which we assume to be a function of window means. We define the covariance matrix for condition *j *in window *I as*

(3) $${\hat{\sum }}_{j}^{\left(i\right)}=\text{diag}\left({\hat{\sigma }}_{j}^{\left(i\right)}\right)\times {\hat{R}}_{j}\times \text{diag}\left({\hat{\sigma }}_{j}^{\left(i\right)}\right)$$

where $\text{diag}\left({\hat{\sigma }}_{j}^{\left(i\right)}\right)$ denotes a diagonal matrix in which the diagonal entries are the estimated standard deviations for the *P *variables specific to window *i*. We denote the diagonal entries as

(4) $${\hat{\sigma }}_{j}^{\left(i\right)}=\left({\hat{\sigma }}_{j1,}^{\left(i\right)}{\hat{\sigma }}_{j2,\ldots ,}^{\left(i\right)}{\hat{\sigma }}_{jP}^{\left(i\right)}\right)$$

To estimate the standard deviation ${\hat{\sigma }}_{jp}^{\left(i\right)}$ for each of the *P *variables in condition *j*, we assume that the variances of replicates of the same factor are identical, and thus estimating ${\hat{\sigma }}_{jp}^{\left(i\right)}$ for variables *p *= 1... *P *reduces to estimating ${\hat{\sigma }}_{j,k}^{\left(i\right)}$ for factors *k *= 1...*K*.

For each factor *k *in each condition *j*, we calculated the replicate mean ${\bar{x}}_{j,k}^{\left(i\right)}$ and the unbiased estimate of the variance ${\sigma_{j,k}^{2}}^{\left(i\right)}$ of replicates in each window *i*. Due to possible correlation between replicates, the commonly used sample variance estimator may under estimate the true variance. Instead, we used the following de-correlated values to estimate the replicate variance ${\sigma_{j,k}^{2}}^{\left(i\right)}$:

$${\left(x_{j,k}^{\left(i\right)}-{\bar{x}}_{j,k}^{\left(i\right)}\right)}^{\prime }*\text{Chol}\left({\hat{R}}_{j,k}\right)$$

Here $x_{j,k}^{\left(i\right)}$ is a vector of length *R *and ${\hat{R}}_{j,k}$ denotes a sub-correlation matrix for replicates of factor *k *in condition *j*, obtained from the full correlation matrix ${\hat{R}}_{j}$. Chol(.) denotes Cholesky decomposition.

We fitted a LOESS curve to the (${\bar{x}}_{j,k}^{\left(i\right)}$, ${\sigma_{j,k}^{2}}^{\left(i\right)}$) pair of all windows using the same LOESS setting described previously in the normalization section. The fitted smooth curve represents a function between replicate mean and variance. At each window *i*, we estimated ${\hat{\sigma }}_{j,k}^{\left(i\right)}$ by the square root of the fitted value given by the curve at ${\bar{x}}_{j,k}^{\left(i\right)}$. We do this for every factor *k *in every condition *j *to generate $\text{diag}\left({\hat{\sigma }}_{j}^{\left(i\right)}\right)$ in equation (3)

### Log likelihood ratio tests

#### Test 1. Detect binding versus non-binding

We first detect binding events of any factor in any condition. We performed a log likelihood ratio test on the null hypothesis *H_0_*:

(6) $$H_{0}:u_{1}^{\left(i\right)}=\ldots =u_{c}^{\left(i\right)}=u_{bck}^{\left(i\right)}$$

against the alternative hypothesis *H*_1_: at least one $u_{j}^{\left(i\right)}$ differs from the background mean$u_{bck}^{\left(i\right)}$. Both $u_{j}^{\left(i\right)}$ and $u_{bck}^{\left(i\right)}$ denote *P*-dim vectors of means, and we estimated $u_{bck}^{\left(i\right)}$ as the median signals across all conditions in the ±5kb neighbourhood of window *i*, for each factor respectively.

Under a multivariate normal distribution, the MLE ${û}_{j}^{\left(i\right)}$ in condition *j *can be obtained by

(7) $${û}_{j}^{\left(i\right)}=A{û}_{j}^{A\left(i\right)}$$

where  A denotes a *P *by *K *matrix of 0s and 1s. For each column *k = 1,..., K*, there are *R *1s in rows (*k*-1)*r*+(1,..., *R*). ${û}_{j}^{A\left(i\right)}$ is a column vector of length *K *containing the means for *K *factors. Derivations of the MLE of ${û}_{j}^{\left(i\right)}$ in equation (7) can be found in Additional File 1.

We then fit the parameters obtained previously ($u_{bck}^{\left(i\right)},{û}_{j}^{\left(i\right)},{\hat{\sum }}_{j}^{\left(i\right)}$) to multivariate normal distribution and computed maximum of the likelihoods$L_{0}^{\left(i\right)},L_{1}^{\left(i\right)}$ under the two assumptions $H_{0},H_{1}$ by

(8) $$L_{0}^{\left(i\right)}=\sum_{j=1}^{c}\left\{\frac{-p}{2}ln\left(2\pi \right)-\frac{1}{2}ln\left|{\hat{\sum }}_{j}^{\left(i\right)}\right|-\frac{1}{2}{\left(x_{j}^{\left(i\right)}-{û}_{bck}^{\left(i\right)}\right)}^{\prime }{\hat{\sum }}_{j}^{\left(i\right)-1}\left(x_{j}^{\left(i\right)}-{û}_{bck}^{\left(i\right)}\right)\right\}$$

(9) $$L_{1}^{\left(i\right)}=\sum_{j=1}^{c}\left\{\frac{-p}{2}ln\left(2\pi \right)-\frac{1}{2}ln\left|{\hat{\sum }}_{j}^{\left(i\right)}\right|-\frac{1}{2}{\left(x_{j}^{\left(i\right)}-{û}_{j}^{\left(i\right)}\right)}^{\prime }{\hat{\sum }}_{j}^{\left(i\right)-1}\left(x_{j}^{\left(i\right)}-{û}_{j}^{\left(i\right)}\right)\right\}$$

Consequently, the standard likelihood ratio is given by $\text{ln}\left(\frac{L_{0}^{\left(i\right)}}{L_{1}^{\left(i\right)}}\right)$. Then we can compute the likelihood ratio test statistic $T^{\left(i\right)}$ for the null hypothesis (6) for window *i *by

(10) $$T^{\left(i\right)}=-2\text{ln}\left(\frac{L_{0}^{\left(i\right)}}{L_{1}^{\left(i\right)}}\right)=2\left(L_{1}^{\left(i\right)}-L_{0}^{\left(i\right)}\right)$$

Under the null hypothesis that there are no binding events in window *i *of all factors in all conditions, $T^{\left(i\right)}$ follows an asymptotic Chi-square distribution with *CK *degrees of freedom. Note that this test is bi-directional, i.e., both enriched and depleted binding signals relative to the background can be detected. To detect enriched signals only, we can replace $x_{j}^{\left(i\right)}-{û}_{j}^{\left(i\right)}$ by 0 whenever it is negative.

#### Test 2. Detect differential binding versus common binding

To identify potential regulatory differential binding regions across conditions, we performed a 2^nd ^LRT test on the null hypothesis *H_0_*:

(11) $$H_{0}:u_{1}^{\left(i\right)}=\ldots =u_{c}^{\left(i\right)}=u_{0}^{\left(i\right)}$$

against the alternative hypothesis *H*_1_: at least one $u_{j}^{\left(i\right)}$ differs from the rest, where $u_{0}^{\left(i\right)}$ denote the averaged mean signals across conditions within window *i *(as opposed to the ±5kb neighbourhood around window *i *in the 1^st ^test). While we used the same values of $u_{j}^{\left(i\right)}$ as used in the 1^st ^test, we estimated the MLE ${û}_{0}^{\left(i\right)}$ by

(12) $${û}_{0}^{\left(i\right)}=A{û}_{0}^{A\left(i\right)}$$

Again, ${û}_{0}^{A\left(i\right)}$ is a column vector of length *K *containing the means for *K *factors, and derivations of the MLE of ${û}_{0}^{A\left(i\right)}$ in equation (12) can be found in Additional File 1. The LRT statistic for the null hypothesis (11) has the same form as (10), but with $u_{bck}^{\left(i\right)}$ replaced by ${û}_{0}^{\left(i\right)}$ in (8) and (9). Under the null hypothesis that the binding (or non-binding) signals for all factors are consistent across conditions in window *i*, the test statistic follows an asymptotic Chi-square distribution with (*C-1)K *degrees of freedom.

#### Test 3. Identify condition specific binding

The p-values of our log likelihood ratio tests are given by the tail probabilities of the chi-square distribution with *CK *degrees of freedom for the 1^st ^test and (*C*-1)*K *degrees of freedom for the 2^nd ^test, respectively. To call statistically significant regulatory regions (1^st ^test) and differential binding regions (2^nd ^test), we used Bonferroni correction to adjust p-values and then used 0.05 as the cutoff. Only the windows rejected by the 1^st ^test are further tested by the 2^nd ^test. After the 2^nd ^test calling significant regions that have at least one condition showing differential binding in at least one factor, we further want to evaluate which regions have differential binding in one and only one condition. To check if the differential binding event in window *i *occurs only in condition *j*, we applied a 3rd test, which is equivalent to the 2^nd ^test but leaving out the data in the current condition *j*, for all *j *= 1 ... *C*. We obtained another statistics $T_{-j}^{\left(i\right)}$ based on the log likelihood ratios using data in only *C*-1 conditions.

(13) $$T_{-j}^{\left(i\right)}=2\left(L_{0,-j}^{\left(i\right)}-L_{1,-j}^{\left(i\right)}\right)$$

The $T_{-j}^{\left(i\right)}$ statistic can be treated as the deviance obtained by the other conditions while leaving out *j*. Since we already know that window *i *has differential binding signals, we can decide if the differential binding event only occurs in condition *j *by checking the significance of $T_{-j}^{\left(i\right)}$. In particular, we say that window *i *has differential binding only in condition *j *if $T_{-j}^{\left(i\right)}$ is the only statistics, compared to the statistics for other conditions, with adjusted p-value > 0.05 in window *i *(i.e., insignificant only by removing the data from *j*).

### Data simulation

To evaluate the performance of our method, we applied dCaP to a simulated dataset containing 30 data tracks including 5 conditions, 3 factors in each condition, and two replicates per factor. Each data track consists of 30,000 data points. Since the variances of the log_2 _signals in the non-binding and binding regions in the human ENCODE data set were about 4 and 1, respectively, we used the same values in the simulation. For each data track, non-binding background signals were sampled independently from a normal distribution with mean 0 and variance 4. We then randomly selected 1,800 windows as binding regions in any of the five conditions, and we simulated the binding signals from a normal distribution with mean 8 and variance 1. Among the 1,800 binding windows, 1,200 were simulated as common bindings that appeared in all five cell lines and three factors. For the remaining 600 bindings, we randomly assigned an integer *M *(0<*M<5*) to each window and assigned binding signals to all three factors in *M *out of the 5 conditions. As a result, these 600 bindings can be treated as differential bindings. Correlations among the three factors were also simulated to mimic those observed in the human ENCODE data. In addition to the simulated data described above, we generated two more datasets in the same setting except that we changed the mean and variance of the binding signals to (6, 1.56) and (4, 2.25), respectively. Accordingly, we call the three simulated datasets strong, medium and weak binding data, respectively. As Negative Binomial distribution was found to provide better fit to the count data in ChIP-seq experiments [34], we conducted another simulation using data from Negative Binomials. Details can be found in Additional File 2.

## Results

### Simulation study

**Figure 2:**
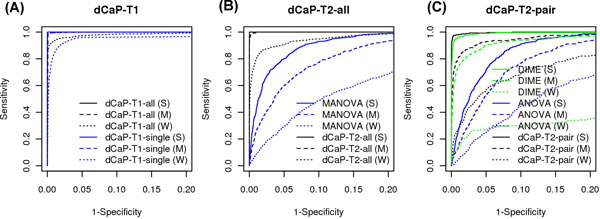
**The ROC curves generated using simulated data at different levels of binding strengths (strong, medium, weak)**. (A) ROC curves of dCaP-T1-all and dCaP-T1-single. (B) ROC curves of dCaP-T2-all and MANOVA (C) ROC curves of dCaP-T2-pair, DIME and ANOVA.

Using the simulated data described in Methods, we first evaluated the performance of dCaP Test 1 for joint peak calling. We compared the results of dCaP Test 1 using 5 conditions together (dCaP-T1-all) with the union of the results obtained from each condition separately (dCaP-T1-single). Figure 2A shows the Receiver Operating Characteristics (ROC) curves generated by dCaP-T1-all and dCaP-T1-single for the three data sets at different levels of binding strengths (strong, medium, weak). We found that the performance of dCaP-T1-all is consistently better than dCaP-T1-single at all levels of binding strengths, indicating the power gain of joint peak calling versus separate peak calling. Particularly, the power of dCaP-T1-all (PRC 94.5%) is substantially better than the merged results from dCaP-T1-single (PRC 85.8%) for the weak binding data (dotted black and blue lines in Figure 2A).

We next evaluated the performance of dCaP Test2 (dCaP-T2-all) for detecting differential binding of multiple factors in multiple conditions. We compared dCaP-T2-all to multivariate analysis of variance (MANOVA) and the resulting ROC curves are shown in Figure 2B. Again, we observed that dCaP-T2-all is substantially more powerful than MANOVA at all levels of binding strengths. We further compared the performance of dCaP-T2 in a two-sample-one-factor scenario (dCaP-T2-pair) with ANOVA and DIME [18]. The ROC curves (Figure 2C) again show that dCaP-T2-pair performs consistently better than the other two methods at all levels of binding strengths, and the performance of DIME is better than ANOVA in the strong and weak binding data. In addition to using simulated multivariate normal data, we also compared the performance of dCaP-T2-all with the above methods (ANOVA/MANOVA/DIME) along with DBChIP using simulated Negative Binomial data. Except for MANOVA/dCaP, the output statistics for other methods were obtained from each pairwise comparison on a single factor between all pairs of conditions. After combining the pairwise statistics using Fisher's method, we used ROC curve to compare their performance. Detailed methods can be found in Additional File 2. Our findings showed that while dCaP-T2-all, DIME, and DBChIP outperformed MANOAVA/ANOVA at all levels of binding strengths, these three methods performed equally well at the strong and medium binding sites. For the weak binding sites, dCaP-T2-all outperformed the others (Additional File 2 Figure S1).

### Application to human ENCODE Data

**Figure 3:**
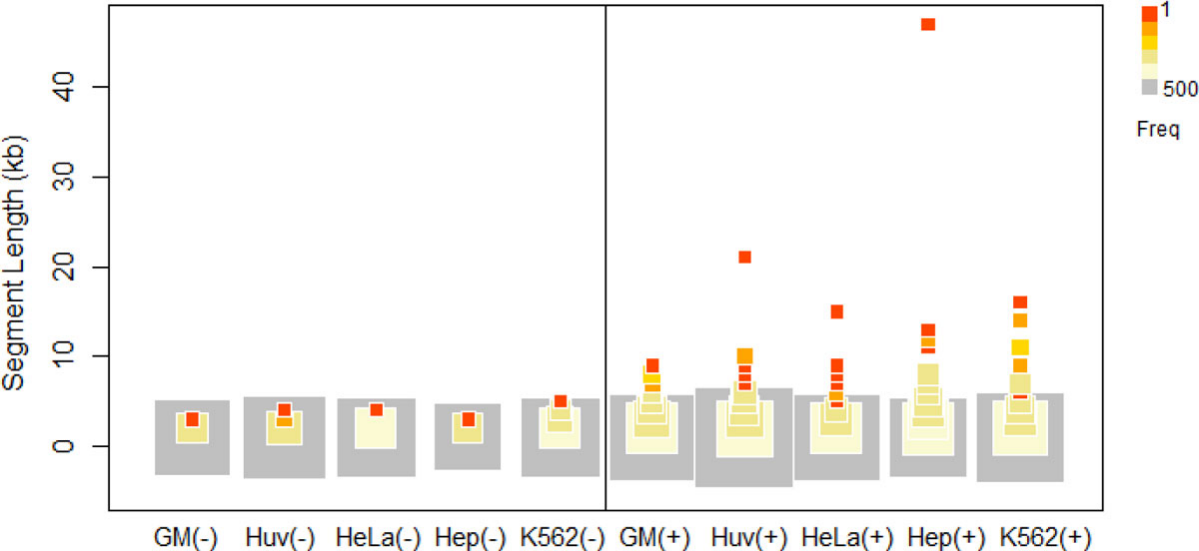
**Length distribution of cell-type specific enriched binding (+) and depleted binding (-) segments**. The symbol size is proportional to the frequency of the corresponding segment length.

By applying dCaP to the ENCODE data sets of three epigenetic factors (CTCF, Pol2, DNaseI) in five cell lines (GM12878, HUVEC, HeLa-S3, HepG2, K562), we detected 194,840 (6.9% of all regions tested) significant occupancy regions genome-wide (excluding chromosome Y) at Bonferroni adjusted 0.05 significance level. Among those significant regions, 33,205 (17% of 194,840) showed differential signals of the 3 factors among the 5 cell lines. Furthermore, 16,452 (49% of 33,205) of the differential regions were cell-type specific. After merging the contiguous regions, we obtained 13,858 cell-type specific intervals. A majority of cell-type specific intervals have length 1-kb. Yet, we found a set of signal-enriched regions with length longer than 10 KB (Figure 3). These long cell-type specific intervals were mostly due to the long span of POL2 signals, and they are consistent with the cell-type specific gene expression (ENCODE RNA-Seq). Examples of these regions include genes *ASHG, AFP, GPC3 *in HepG2 cell line, *THBS1 *in HUVEC cell line, *BC068609, LOC648232 *and a 30 KB region close to 7q36.2 (starting from position 152,710,000 in hg18) in K562 cell line. We also observed clusters of cell-type specific depleted signals in local intervals, which were mostly due to large genomic deletions.

**Figure 4:**
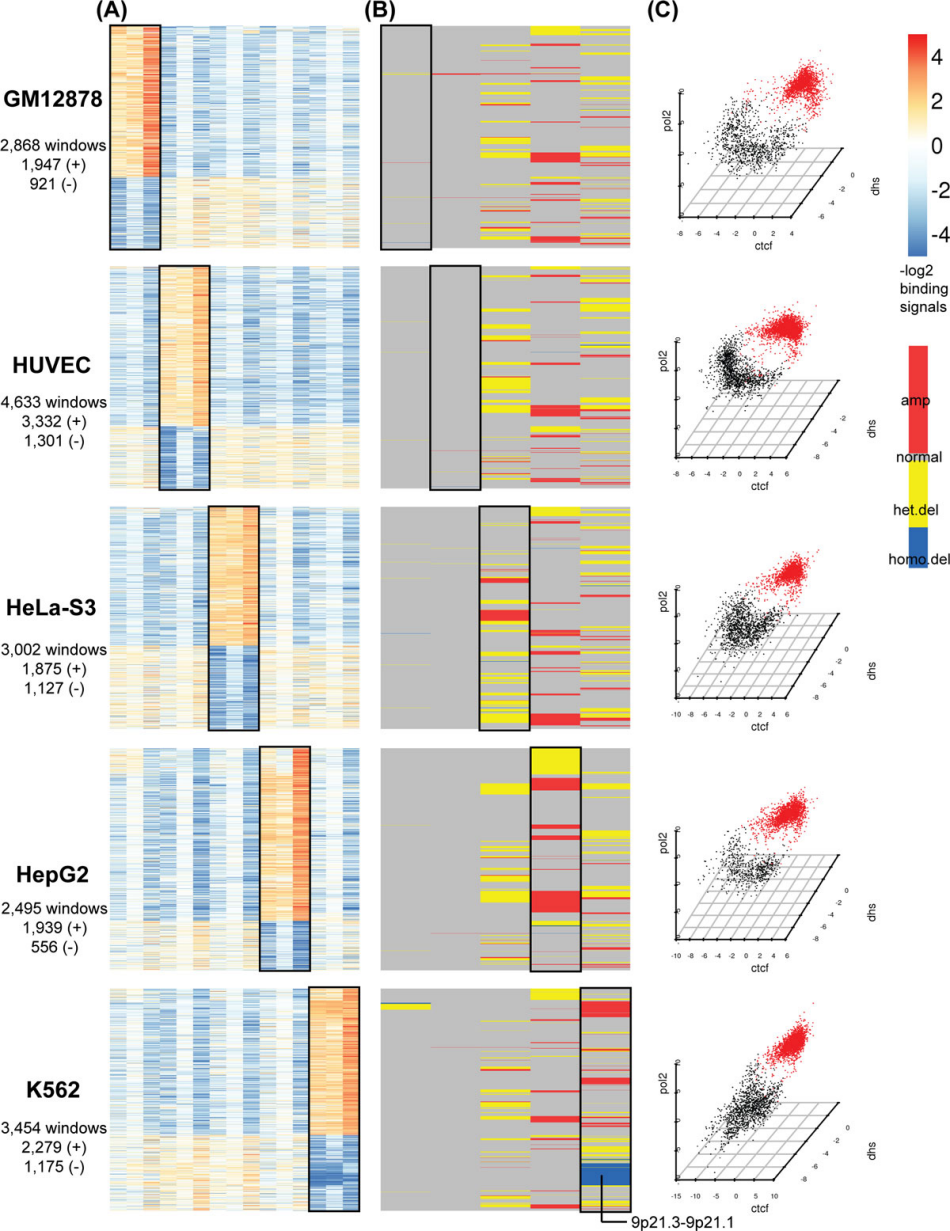
**Patterns of binding and CNV in the five groups of cell-type specific regions identified by dCaP**. (A) The five heatmaps represent the five groups of cell-type specific regions identified by dCaP. Each heatmap corresponds to one specific cell-type. Each row in a heatmap shows a cell-type specific region and each column shows the relative binding signals from one of the 15 data tracks. The first three columns are the relative binding signals of the three factors (CTCF, DHS and POL2) in the GM12878 cell lines. The remaining columns are ordered in the same way for HUVEC, HeLa-S3, HepG2 and K562 cell lines, respectively. The rows are first ordered by their clustering class (enriched/depleted) and within each class, the cell-type specific regions are ordered by their genomic locations. (B) Heatmap of CNVs in the five groups of cell-type specific regions. The rows of the heatmap are arranged in the same way as in A and the columns show different types of CNVs in order of GM12878, HUVEC, HeLa-S3, HepG2 and K562. (C) Scatter plots of K-means clustering on the relative binding signals of the three factors in each group of cell-type specific regions.

To explore the binding patterns in each group of the cell-type specific regions, we centred the binding signals of the 3 factors by their means across all cell types. We then used these relative binding signals to perform K-means clustering to the cell-type specific windows to identify co-occupancy patterns of the 3 factors in the corresponding cell types. We varied the number of clusters K from 2 to 12 and chose the optimal K using the Calinski-Harabasz index, which evaluates the cluster validity based on the average between- and within-cluster sum of squares [35]. The clustering results showed that each of the five groups of cell-type specific windows mainly demonstrated two distinct patterns: one with enriched signals of all 3 factors in the corresponding cell, and the other with consistently depleted signals. To visually examine the epigenetic signals in each group of cell-type specific regions in each cluster, we generated heatmaps of relative binding signals across cell types, their corresponding copy number variations (CNVs, ENCODE common cell CNV, HAIB Genotype Track [36]), and scatter plots, in Figure 4 (A,B,C), respectively.

#### Cell-type specific depletion in tumor cell lines is associated with CNVs

DNA copy number variation is a common genetic alteration observed in solid tumors, and it contributes to tumor evolution by alterations of the expression of genes within the region [37]. As shown in Figure 4 (A, B), compared to the normal cell lines, the cell-type specificity in the cancer cell lines (HeLa-S3, HepG2 and K562) is strongly associated with copy number variations of various kinds (amplified, heterozygous deletion or homozygous deletion) [36]. There are many more CNVs in the cancer cell lines than in the two relatively normal cell lines. K562 has the most CNVs with a genome-wide coverage of 715 Mb, and the CNV coverage of HeLa-S3 and HepG2 are around 613 Mb and 550 Mb, respectively. In contrast, the CNV coverage in the two normal cell lines are both <5 Mb.

Consistent with the genome-wide CNV distribution, 37% of the K562-specific binding windows co-occurred with the CNVs, which accounted for the most CNV overlap among the 5 groups of cell-type specific windows. Among the 5 cell types, K562 also had the largest number of depleted binding windows (364, 90.77%) overlapping with homozygous deletions (Figure 4B). As expected, homozygous deletions were only found in the cell-type specific non-binding regions, but not in the enriched regions. On the other hand, heterozygous deletions were found in both enriched and depleted cell-type specific regulatory regions. In HeLa-S3 and K562 cell lines, heterozygous deletions occurred 2-3 times more frequently in the depleted binding regions than in the enriched ones. Conversely, there were 5 times more heterozygous deletions in HepG2-specific enriched binding regions than in the depleted ones.

**Figure 5:**
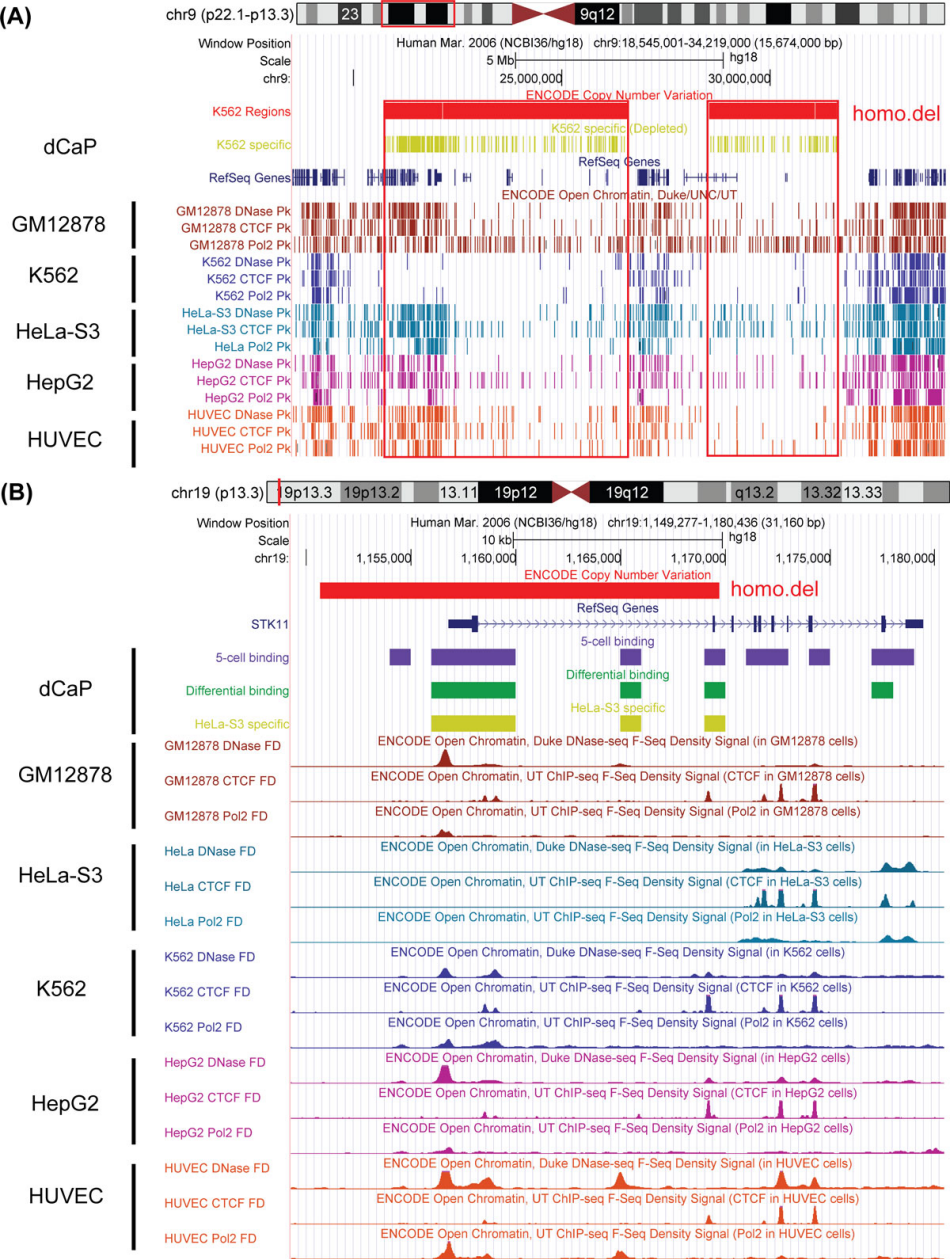
**Examples of cell-type specific regions detected by dCaP**. (A) K562-specific depleted binding detected by dCaP (shown in olive) associated with large homozygous deletions on chromosome 9. (B) HeLa-specific depleted binding detected by dCaP close to the TSS of gene STK11 (binding windows shown in purple, differential binding in green and cell-type specific windows in olive).

Figure 5A shows an example of 342 K562-depleted windows clustered (shown in olive) in a 10.8-MB region on 9p21.3-9p21.1, which were associated with 8 homozygous deletions. These homozygous deletions spanned approximately 9-MB and the loss of DNA sequence in this region has been previously observed in solid tumors and leukemia [38,39]. Many genes in this region (e.g., the *CDKN2A/CDKN2B *locus and *MTAP*) were found to be associated with different types of cancer [40-42]. Owing to this large deletion, we observed consistent depletion of CTCF, DHS, POL2 signals in K562. In contrast, ENCODE called a few binding peaks in K562 cell lines (shown in dark blue in Figure 5A) in this homozygous deletion region, which are likely false positives.

Figure 5B shows another example of depleted cell-type specific binding in HeLa-S3, which is located in gene locus *STK11/LKB1 *with tumor suppressor function [43]. We see that, in gene *STK11*, 7 binding regions (in purple), 4 differential binding regions (in green) and 3 cell-type specific differential binding regions (in olive) were detected by dCaP. One HeLa-S3 specific depleted binding region (in olive) was observed near the transcription start site (TSS) of *STK11 *and was found to be associated with a homozygous deletion highlighted in red (Figure 5B). Furthermore, we observed loss of RNA-Seq signals (ENCODE Caltech RNA-Seq) in the first three exons of *STK11 *in HeLa-S3 cell lines (the last track of Figure 5B), which coincided with the three cell-type specific differential binding regions detected by dCaP. A recent study (29) reported that *STK11 *plays an important role in multiple cellular functions including cell growth, cell cycle progression, metabolism, cell polarity, and migration. *STK11/LKB1 *is a tumor suppressor gene with lower expression in HeLa cell lines (cervical cancer cells) [43]. Our results suggest that the cell-type specific depleted binding in cancer cell lines may be due to genomic deletions. The cell-type specific differential binding regions detected by dCaP in cancer cell lines can pinpoint the regulatory loci that may undergo genetic alterations, and hence their nearby target genes may be the potential candidate genes associated with cancer.

#### Cell-type specific enriched binding are enriched for cell-type specific functions

**Figure 6:**
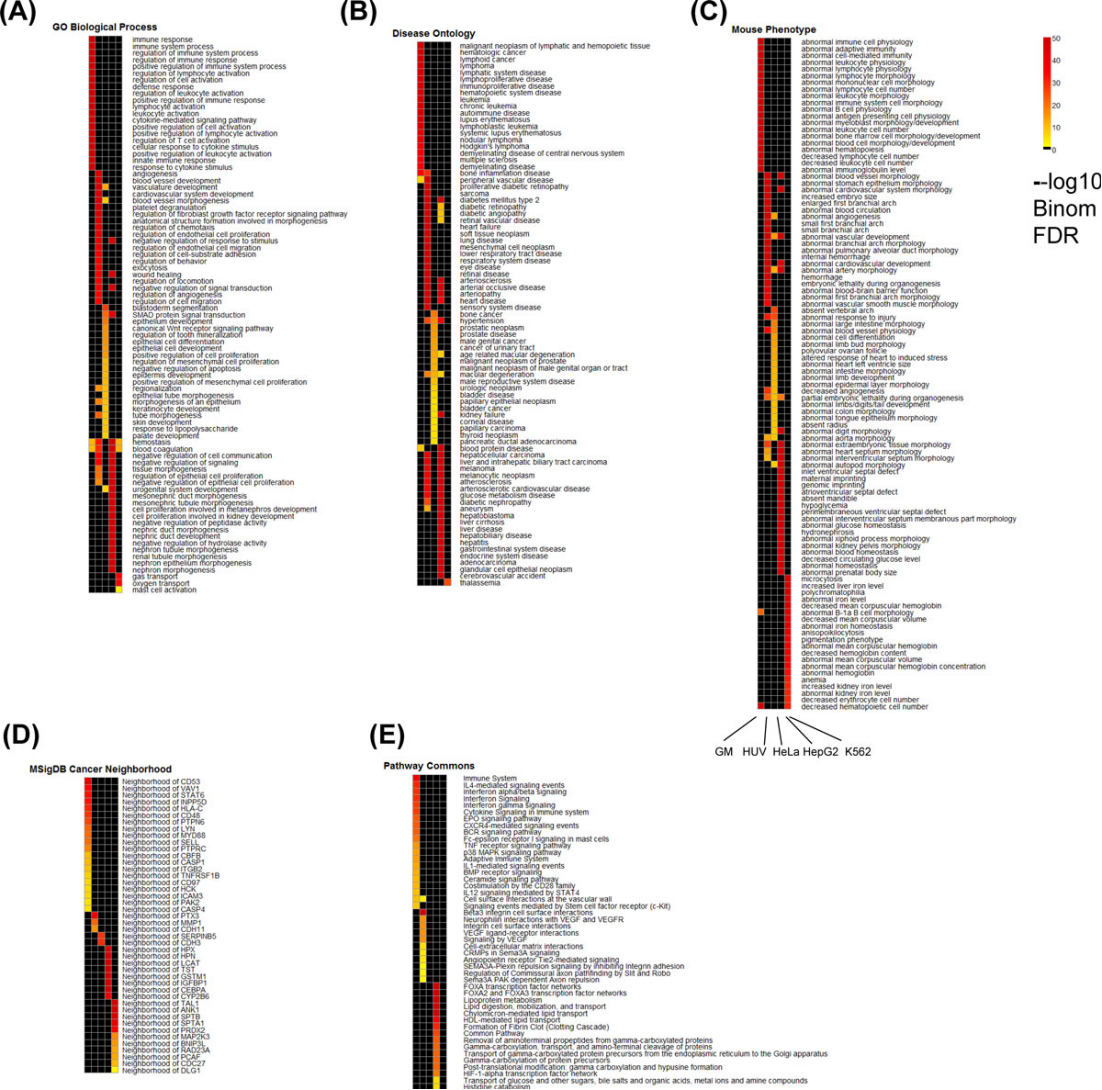
**Functional enrichments from GREAT in the enriched binding cluster of cell-type specific windows**.

The functional categories detected using GREAT version 2.0.2 (http://great.stanford.edu) [44] revealed that our cell-type specific intervals of enriched signals were significantly associated with genes carrying cell-type specific functions. We show the top 20 significant functional terms in Gene Ontology (GO), Disease Ontology, Mouse Phenotypes, Cancer Neighbourhoods and Pathway Commons in Figure 6. We associated each cell-type specific binding window to its single nearest gene up to 1 Mb and required that the significant terms satisfy both Binomial and Hypergeometric FDR <5% and region fold enrichment >=2. The -log_10 _Binomial FDRs were used to generate the heatmap of the function annotation terms, where black denotes no significant terms found by the above criteria. Below we elaborate the enrichment analysis results for the cell-type specific binding regions in each of the five cell lines, which demonstrate strong evidence that dCaP can pinpoint the genomic loci that play important roles in regulating cell-type specific functions and diseases.

GM12878 (transformed human B-lymphocyte cell line): GM12878-specific enriched (GM-enriched) binding windows were found to be near genes with GO terms specially in immune response, regulation of immune system process, regulation of cell/lymphocyte/leukocyte/T-Cell activation, defense response, cellular response to cytokine stimulus and cytokine mediated signaling pathway (Figure 6A). In addition, the GM-enriched windows were also close to genes in MHC class II receptor activity, SH3 domain binding (GO Molecular Function). An enrichment analysis of Disease Ontology highlights many lymphoid diseases, such as lymphoid cancer, lymphoma, lymphoblastic leukemia and also other auto-immune diseases (lupus erythematosus) (Figure 6B). Similar lymphoid/immune related terms were also reported in Mouse Phenotypes (Figure 6C) and were highly related to the cell type of GM12878. From the MSigDB Cancer Neighbourhood Ontology, we found that the GM-enriched binding windows were in proximity to cancer-associated genes, *CD53, VAV1, INPP5D, STAT6, HLA-C*, etc (Figure 6D). GREAT also reported GM-enriched windows involved in T cell/B cell activation (Panther Pathway), and GM-enriched windows were close to genes involved in BCR, TCR, PD-1 signaling, intestinal immune network for IgA production, costimulation by the CD28 family, antigen processing and presentation, etc (MsigDB Pathway). There were also other pathways specific to GM12878, for examples, cytokine signaling in immune system and interferon alpha/beta/gamma signaling pathway (Figure 6E). An enrichment analysis of HGNC Gene Families showed that the GM-enriched windows were close to members of the *HLA *and *CD *family. The InterPro Ontology also reported that genes with immunoglobulin/major histocompatibility conserved site, MHC class I/II antigen recognition protein domain, and SH2 motif, were close to GM-enriched windows. The Predicted Promoter Motifs ontology reveals that the motifs of SPI1, ETV4, ELF1, IRF1 (IFN-regulatory factor-1), RUNX1, ETV7, STAT1 (a key transcription factor in the interferon signaling pathway), and NFKB, were found in the promoters of genes associated with GM-enriched windows. This result suggests that the above TFs could be potential co-regulators of genes near GM-enriched windows.

HUVEC (umbilical vein endothelial cell line): The enriched GO terms for HUVEC-enriched binding windows were also relevant to its tissue type. Targets of the HUVEC-specific enriched windows were found to be enriched in angiogenesis, blood vessel, vasculature development, blood vessel morphogenesis, and regulation of endothelial cell migration, etc (Figure 6A). The disease ontologies enriched in genes near HUVEC-enriched windows highlighted many vascular-related diseases, including arteriopathy, arteriosclerosis, diabetic angiopathy, eye diseases (diabetic retinopathy) and heart diseases (Figure 6B). The InterPro ontology also reported genes with the epidermal growth factor-like type 3, EGF-like conserved site near HUVEC-enriched windows. Enrichments from the Pathway Commons ontology highlighted Beta3 integrin cell surface interactions, VEGF specific signaling, and many others (Figure 6E).

HeLa-S3 (cervical epithelial carcinoma cell line): The top enriched GO terms for the HeLaS3-enriched windows were associated with HeLa-S3's epithelial cell type, such as epithelium development, epithelial cell differentiation/development and skin development. Other terms including SMAD protein signal transduction and canonical Wnt receptor signaling pathway were also found to be enriched around the HeLa-enriched windows. Besides, a few GO terms and mouse phenotypes related to vessel development (vasculature development, blood vessel morphogenesis, abnormal angiogenesis, etc) were shared between HeLa-S3, HUVEC, and HepG2 (Figure 6A, 6C). Since HeLa-S3 is derived from a human cervix adenocarcinoma, compared to GM12878 and HUVEC, we found that many enriched disease phenotypes detected by GREAT in this group were related to different types of cancer, including bone, prostatic, urinary tract, pancreatic cancer (Figure 6B). Among those, papillary epithelial carcinoma was found strongly associated to HeLa-S3.

**Figure 7:**
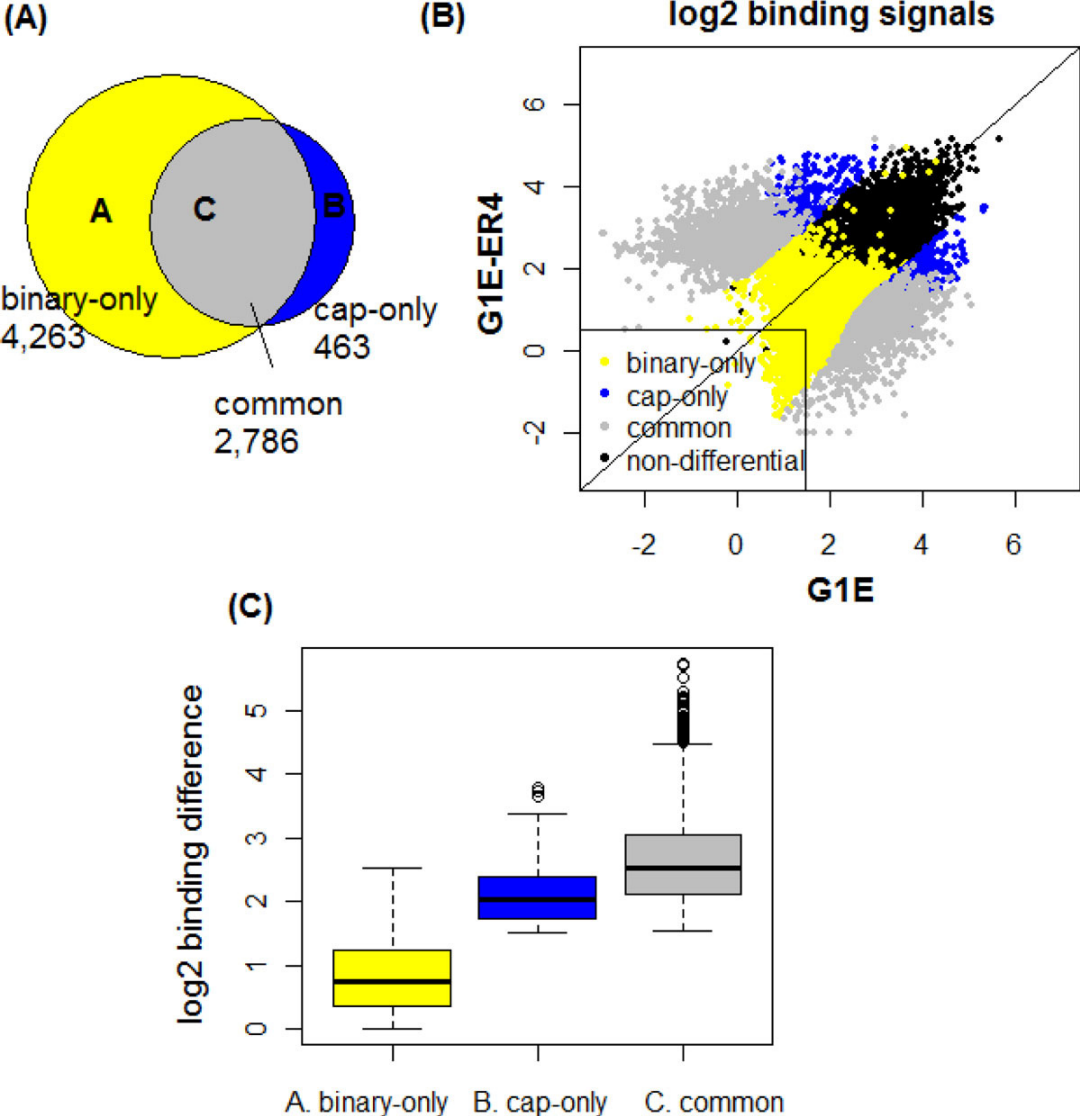
**Comparison of differential binding detected by intersection of binary peaks and dCaP**. (A) Venn diagram of TAL1 DPs in the three categories. (B) Scatter plot of the log2 TAL1 binding signals in the merged binding peaks (C) Boxplot of the absolute binding difference in G1E and G1E-ER4 cell lines for the three groups of DPs.

HepG2 (hepatocellular carcinoma cell line): The HepG2 cell line is derived from a male with a well differentiated hepatocellular carcinoma. Among the 5 cell types, we observed that HepG2 has the largest number of enriched binding windows (494, 41.24%) in the amplification CNVs. The enriched GO terms of genes close to the HepG2-enriched windows were unique from the other cell types. Top enriched GO terms (Molecular Function) showed enrichment in peptidase inhibitor/regulator activities, enzyme inhibitor activities, and endopeptidase inhibitor/regulator activities. The GO terms in Biological Process show more detailed terms in regulation in the above activities and also including terms relevant to kidney development, such as mesonephric tubule morphogenesis. (Figure 6A). Most of the top disease terms detected in HepG2-enriched windows are all related to liver diseases, including hepatoblastoma, liver cirrhosis, hepatitis, fatty liver, hepatocellular carcinoma. GREAT also reported that HepG2-enriched windows were close to many cancer genes *HPX, HPN, LCAT, TST, GSTM1, CEPBA, CYP2B6*. (Figure 6D). Unique pathways, such as FOXA2 and FOXA3 transcription factor networks, Lipoprotein metabolism (Pathway Commons) were also reported by GREAT (Figure 6E). Among these pathways, the FOXA1, FOXA2 and FOXA3 transcription factor networks were reported to be expressed early in embryonic endoderm and play important roles in the regulation of gene expression in liver and pancreas [45]. The MsigDB Predicted Promoter Motifs ontology showed that HepG2-enriched windows were close to genes whose promoters contain binding sites for TCF1/HNF1 (hepatocyte nuclear factor 1) and FOXA1/ HNF3α. The Transcription Factor Targets ontology has compiled data from ChIP experiments that link transcription factor regulators to downstream target genes. GREAT revealed that the HepG2-enriched windows were located near genes regulated by master regulators of hepatocyte and islet transcription, such as HNF1α and HNF4α in human liver and pancreatic islets (Binomial FDR<2.4e-34) [46]. Furthermore, the strongest term in MSigDB Perturbation ontology showed enrichment in liver selective genes. Particularly, 16.34% of HepG2-enriched windows were within the neighbourhoods of liver genes. The HGNC Gene Families ontology also indicated that 18 HepG2-enriched windows were located near 13/33 SERPIN gene families.

K562 (chronic myelogenous leukemia cell line): The targets of K562-specific binding windows were enriched in Mouse Phenotypes, such as microcytosis, decreased erythrocyte/hematopoietic cell number, anemia, abnormal homoglobin, abnormal iron hemostasis, etc (Figure 6C). The MSigDB Cancer Neighborhood highlighted cancer-associated genes, *TAL1, ANK1, SPTB, SPTA1, PRDX2, MAP2K3*, etc. (Figure 6D). The MSigDB Perturbation ontology also showed enrichment of the top 40 genes from cluster 7 of acute myeloid leukemia (AML) expression profile in a previous study [47].

### Application to mouse ENCODE data

We further applied dCaP Test 2 (dCaP-T2) to detect differential TAL1 occupancy before and after restoration of GATA1 in two mouse erythroid cell lines (G1E, G1E-ER4) reported in Wu et. al [15]. Since TAL1 and GATA1 are critical erythroid transcription factors and often co-bind during erythroid differentiation, studying the differential binding of TAL1 in these two cell lines allows us to understand the mechanisms of erythroid gene induction and repression by transcription factor occupancy.

Among the 8,002 and 4,915 TAL1 peaks called in G1E and G1E-ER4 cell lines [15], respectively, we obtained 7,049 differential peaks (DPs) of TAL1 as determined by the unique binding sites called in each of the two cell lines (i.e., the binary method). In comparison, we ran dCaP-T2 on the union of TAL1 peaks from the two cell lines. At a FDR 10% threshold, dCaP reported 3,249 DPs of TAL1. We then compared the results between dCaP-T2 and the binary method.

By intersecting the DPs from dCaP and the binary calls, we assigned the DPs into three categories: (A) DPs only called by the binary method (binary-only); (B) DPs only called by dCaP (cap-only); and (C) DPs called by both methods (common). The Venn diagram of these DPs is shown in Figure 7A. There are many more DPs called by the binary method (7,049) than by dCaP (3,249). We observed that 85% of the DPs by dCaP agreed with the DPs by binary intersection, and 40% of the DPs by the binary method agreed with the DPs by dCaP.

To evaluate the TAL1 DPs in the three categories, we generated a scatter plot showing the binding signals of all TAL1 occupancy (Figure 7B) and a boxplot of their absolute binding differences (Figure 7C) between the two cell lines, for each of the three groups of DPs. Figure 7B shows that most of the binary-only DPs are distributed along the diagonal line and many of them tend to have lower binding signals in both cell lines compared to the other two DP groups. Figure 7C further shows that the DPs in the binary-only group have the least binding variation between G1E and G1E-ER4. In fact, most (95%) of the binary-only DPs had TAL1 peaks identified in G1E but not in ER4. When considering the magnitude of binding signals instead of the binary peak calls, however, only 77% of the binary-only DPs showed stronger TAL1 signals in G1E than in ER4. This inconsistency is created by calling peaks in each cell line separately. In addition, around 70% of the binary-only DPs are the weaker binding events with log2 signals <2. Therefore, these DPs were not considered as differential occupancy by dCaP after taking the signal magnitude into account.

In contrast to the binary-only DPs, the cap-only DPs showed significant signal variations. The cap-only DPs were missed by the binary method because they were called as binding in both cell lines. In total, we detected 463 cap-only DPs, which accounted for 16% of TAL1 "common" binding in G1E and G1E-ER4 by the binary method. In contrast to the 77% for binary-only DPs, only 40% of the cap-only DPs had stronger binding signals in G1E than in ER4. As a reference, among the common DPs detected by both methods, 38% of common DPs had stronger signals in G1E than in ER4. Therefore, the percentage of cap-only DPs is very close to percentage observed in the common DPs. In addition, the binding variation of cap-only DPs is much larger than that of the binary-only DPs, and is similar to the binding variation of common DPs (Figure 7C).

We further intersected the DPs in the three groups with relevant biological features to explore their functional importance, including features relevant to TAL1 differential occupancy, such as GATA1 binding in G1E-ER4, differentially expressed genes (between G1E and G1E-ER4), SNPs, and features not relevant to TAL1 differential occupancy, such as non-differentially expressed genes and CpG islands. We expect that the true TAL1 DPs should be highly overlapping with the features relevant to TAL1 differential occupancy, but not so with the irrelevant features.

Table 1 summarizes the proportions of TAL1 DPs in the three categories overlapping with different biological features. We found that the proportions of overlaps for cap-only and binary-only DPs are significantly different (fisher's exact test) only in features relevant to TAL1 differential occupancy, such as induced genes, differentially expressed genes, SNPs (dbSNP128), GATA1 binding peaks and GATA1 motifs. In particular, the proportions of overlaps for cap-only DPs are consistently higher than those for the binary-only group. The unequal proportions of overlaps among groups are unlikely to be random, especially because there is no significant difference in the proportions of overlaps for features irrelevant to the TAL1 DPs (e.g. non-differential expressed genes and CpG islands). These findings suggest that dCaP is more accurate in detecting true TAL1 differential occupancy than the binary method.

**Table 1:** Proportions of TAL1 differential binding in the three categories overlapping with relevant biological features.

	A. binary-only	B. cap-only	C. common
**induced genes^1 ^**	18.77%	*** 32.83%	26.20%
**repressed genes^1 ^**	9.85%	9.94%	6.89%
**all differential expressed genes^1 ^**	28.62%	*** 42.76%	33.09%
**non-differential expressed genes^1 ^**	52.08%	49.46%	50.75%
**GATA1 binding peaks^2^**	36.03%	*** 95.68%	63.17%
**GATA motifs^3^**	66.27%	*** 86.61%	59.51%
**CpG islands^4^**	2.39%	3.67%	2.19%
**SNPs^4^**	84.52%	** 89.42%	82.23%

Significance of difference in proportions between group A and B is denoted by * for each biological feature examined. ** denotes p-values between 5e-2 and 5e-3. *** denotes p-values < 5e-3. The p-values were obtained from the Fisher's Exact Test. The differential expressed genes in G1E and G1E-ER4 were obtained from the transcriptome data in G1E and G1E-ER4 cells^1 ^[15]. GATA1 binding peaks were obtained from [15]. GATA motifs^3 ^were identified by MEME relevant tools [49]. Annotation data such as CpG islands^4 ^and SNPs^4 ^(dbSNP 128) were from database of UCSC genome browser [50].

In summary, our results indicate that it is important to consider quantitative variation when studying differential regulation. The cap-only DPs were not detected by the binary method because they were called as peaks in both cell lines, despite of the fact that they demonstrated significant signal variation that may have important indication of biological functions.

## Conclusions

We have proposed a new powerful method called dCaP that incorporates the quantitative information of multiple epigenetic features to detect both constitutive and variational protein-DNA interaction events in multiple conditions. Given that epigenetic features are highly correlated, the combinatorial effects of multiple proteins may only be detectable using a joint approach, i.e., the signal variation of one factor may only be observable by conditioning on the data of other factors. The variance of binding signals is frequently heterogeneous for different factors at different genomic positions. To gain power, we estimated the variances of noise as a function of the means within each pair of condition and factor using local smoothing techniques. We explicitly used replicates to estimate binding variances. If there are no replicates, we can utilize data across conditions to estimate the variances for each factor. We then designed a 3-step log likelihood ratio test to detect joint, differential, and condition-specific events in a multi-cellular context. The log likelihood ratios generated in each condition can also be used to quantify the contribution of each condition to the total variations. When binding regions are available, instead of running all 3 tests, one can directly apply dCaP-Test2 and dCaP-Test3 on the binding peaks to test the differential and condition-specific binding.

In an application to human ENCODE data set, we detected cell-type specific regulatory regions that mainly demonstrated two patterns: all signals are either enriched or depleted. In general, this may depend on the factors being studied. The cell-type specific enriched regions were strongly associated with functional terms, pathways and diseases relevant to the corresponding cell types. In contrast, the cell-type specific depleted regions, particularly in cancer cell lines, were strongly associated with the CNVs. Among those depleted regions overlapping with homozygous deletions, approximately 97.8% were from the three cancer cell lines, and over 90.7% were found in K562 alone. Similarly, 99.6% of the enriched regions with amplification CNVs were in the three cancer cell lines, with 40.9 % in HepG2, 40.3% in K562, and 18.28% in HeLa-S3. Our results suggest that the cell-type specific depleted signals in cancer cell lines may be under genomic alterations and can be located near genes associated with different types of cancer (i.e. *FHIT, STK11, CDKN2A, CDKN2B*). Other possible causes for the depletion of signals may include genetic mutations, alternative promoters, and epigenetic silencing through methylation that leads to aberrant silencing of normal tumor-suppressor function in cancer cell lines [48]. While we detected many cell-type specific regulatory regions showing consistent binding patterns near a target gene, we also observed some loci near a target gene showing complementary binding patterns. The latter may suggest binding turnover or alternative promoters that are recognized for creating transcript diversity in a cell. To explore the functional consequences of different binding patterns within a local region, future work could incorporate RNA-Seq data into consideration.

In a second application to TAL1 occupancy in response to GATA1 restoration in mouse erythroid cell lines, dCaP detected a unique set of TAL1 differential occupancy sites that were missed by the binary method by Wu et al. (6). Those sites were called in both G1E and G1E-ER4 cells as occupant, yet their binding signals were significantly different. Such quantitative variation is only detectable when considering the magnitude of binding rather than binary presence or absence calls. On the other hand, there were a large portion of "differential binding" called by the binary method alone, which were likely false positives given their small differences in the magnitude of binding between the two cell lines. By further overlapping the DPs with biological features, we observed that the dCaP-only DPs were highly enriched near differentially expressed genes and GATA1 binding sites, while the enrichments for the binary-only DPs were much less significant.

ChIP-seq data is being rapidly generated not only for many TFs and epigenetic marks, but also in multiple conditions, tissues, cell types, and individuals. Using dCaP, both constitutive and variational regulatory regions can be powerfully detected by integrating information in a statistically consistent and efficient way. After finding significant loci of differential regulation and cell-type specificity, the users can intersect the loci with other biological data and functional annotations to derive hypotheses. Clustering analysis of the patterns of signals followed by functional enrichment analysis can also help elucidating the dynamic epigenetic landscapes underlying regulation in multi-cellular contexts. Besides clustering, one can use unsupervised methods like PCA analysis to explore the binding pattern across conditions. PCA loadings can be further used to identify leading factors that contribute most to the difference in the overall binding patterns. As the number of data tracks under comparison increase, directly apply unsupervised methods may not be a good way since they do not provide statistical significance, the increasing noises may confuse clustering results, and PCA does not directly detect differential signals. Our approach is flexible and general. The users can define their own sets of factors and conditions of interest and apply dCaP in a similar way as used in this study. For example, TF binding variations can be detected among individuals by treating each individual as a "condition". Association between epigenetic factors and disease phenotypes can be directly tested by treating groups of individuals with the same phenotype as "conditions" and individuals within groups as "replicates".

## List of abbreviations

ENCODE: encyclopedia of DNA elements; ChIP-Seq: ChIP-Sequencing, chromatin immunoprecipitation followed by massively parallel DNA sequencing; RNA-seq: RNA-sequencing; LRT: log likelihood ratio test; MLE: maximum likelihood estimation.

## Competing interests

The authors declare that they have no competing interests.

## Authors' contributions

KC and YZ conceived and designed the experiments. KC performed the analyses and wrote the manuscript. YZ supervised the study and revised the manuscript. All authors read and approved the final manuscript.

## Declarations

The publication charges of this article were funded by NIH grant R01 HG004718.

## Supplementary Material

Additional file 1 **MLE Derivations of **${û}_{j}^{\left(i\right)}$**and **${û}_{0}^{\left(i\right)}$. Click here for file

Additional file 2 **Simulation using data from negative binomial distributio**n. Click here for file
