# Supplementary material for: dCaP: detecting differential binding events in multiple conditions and proteins
Source: BMC Genomics. 2014 Dec 8;15(Suppl 9):S12. doi: 10.1186/1471-2164-15-S9-S12 (PMC4290593; doi:10.1186/1471-2164-15-S9-S12)
Supplement: Additional file 1 — MLE Derivations of ûjiand û0i. [file 1471-2164-15-S9-S12-S1.pdf]

Derivations of $\hat{\mathbf{u}}_{\mathbf{j}}^{(\mathbf{i})}$ and $\hat{\mathbf{u}}_{\mathbf{0}}^{(\mathbf{i})}$

(i) the MLE of $\hat{\boldsymbol{u}}_j^{(i)}$ in equation (7):

Given $\hat{\boldsymbol{u}}_j^{(i)}$, $\hat{\Sigma}_j^{(i)}$, the log-likelihood $L(\hat{\boldsymbol{u}}_j^{(i)}, \hat{\Sigma}_j^{(i)})$ for cell *j* is obtained using equation (9)

$$L(\hat{\boldsymbol{u}}_j^{(i)}, \hat{\Sigma}_j^{(i)}) = \frac{-p}{2}\ln(2\pi) - \frac{1}{2}ln\left|\hat{\Sigma}_j^{(i)}\right| - \frac{1}{2}(\boldsymbol{x}_j^{(i)} - \hat{\boldsymbol{u}}_j^{(i)})'\hat{\Sigma}_j^{(i)^{-1}}(\boldsymbol{x}_j^{(i)} - \hat{\boldsymbol{u}}_j^{(i)})$$

$$\text{Let } \hat{\boldsymbol{u}}_j^{(i)} = \boldsymbol{A}\,\hat{\boldsymbol{u}}_j^{A(i)}$$

where $\boldsymbol{A}$ is a *P by K* matrix described in equation (7), and $\hat{\boldsymbol{u}}_j^{A(i)}$ is a column vector with length *K* containing the mean for each factor *k.*

For the data we used, we have

$$\boldsymbol{A'} = \begin{bmatrix} 1\,1\,0\,0\,0\,0 \\ 0\,0\,1\,1\,0\,0 \\ 0\,0\,0\,0\,1\,1 \end{bmatrix}$$

Thus, by setting $\frac{\partial}{\partial \boldsymbol{u}_j^{(i)}} L(\hat{\boldsymbol{u}}_j^{(i)}, \hat{\Sigma}_j^{(i)}) = -2\,\boldsymbol{A'}\,\hat{\Sigma}_j^{(i)^{-1}}\left(\boldsymbol{x}_j^{(i)} - \boldsymbol{A}\,\hat{\boldsymbol{u}}_j^{A(i)}\right) = 0$

We get $\boldsymbol{A'}\hat{\Sigma}_j^{(i)^{-1}}\boldsymbol{A}\,(\hat{\boldsymbol{u}}_j^{A(i)}) = \boldsymbol{A'}\,\hat{\Sigma}_j^{(i)^{-1}}(\boldsymbol{x}_j^{(i)})$

We can derive $(\hat{\boldsymbol{u}}_j^{A(i)}) = \left(\boldsymbol{A'}\,\hat{\Sigma}_j^{(i)^{-1}}\boldsymbol{A}\right)^{-1}\left(\boldsymbol{A'}\;\hat{\Sigma}_j^{(i)^{-1}}(\boldsymbol{x}_j^{(i)})\right)$

Then it is trial to obtain $\hat{\boldsymbol{u}}_j^{(i)}$ by $\boldsymbol{A}\,\hat{\boldsymbol{u}}_j^{A(i)}$

(ii) the MLE of $\hat{\boldsymbol{u}}_0^{(i)}$ in equation (12):

Similar to the above derivation, we can set

$$\frac{\partial}{\partial \boldsymbol{u}_0^{(i)}} L(\hat{\boldsymbol{u}}_0^{(i)}, \hat{\Sigma}_j^{(i)}) = \sum_{j=1}^{C} - 2\,\boldsymbol{A'}\,\hat{\Sigma}_j^{(i)^{-1}}\left(\boldsymbol{x}_j^{(i)} - \boldsymbol{A}\,\hat{\boldsymbol{u}}_0^{A(i)}\right) = 0$$

Then we obtain, $\hat{\boldsymbol{u}}_0^{A(i)} = \left(\sum_{j=1}^{C}\boldsymbol{A'}\hat{\Sigma}_j^{(i)^{-1}}\boldsymbol{A}\right)^{-1}\left(\sum_{j=1}^{C}\boldsymbol{A'}\,\hat{\Sigma}_j^{(i)^{-1}}\boldsymbol{x}_j^{(i)}\right)$ and we can get $\hat{\boldsymbol{u}}_j^{(i)}$ by $\boldsymbol{A}\,\hat{\boldsymbol{u}}_0^{A(i)}$
