# Supplementary material for: dCaP: detecting differential binding events in multiple conditions and proteins
Source: BMC Genomics. 2014 Dec 8;15(Suppl 9):S12. doi: 10.1186/1471-2164-15-S9-S12 (PMC4290593; doi:10.1186/1471-2164-15-S9-S12)
Supplement: Additional file 2 — Simulation using data from negative binomial distribution. [file 1471-2164-15-S9-S12-S2.pdf]

Simulation using data from negative binomial distribution

We have shown in the main text that on a task of detecting differential binding while using simulated multivariate normal (MN) data, dCaP not only performed better than MANOVA in the multi-sample-multi-factor scenario (Figure 2B) but also outperformed DIME/ANOVA in the two-sample-one-factor scenario (Figure 2C) at all levels of binding strengths. Here, we investigated the performance of dCaP using simulated negative binomial (NB) data and compared dCaP to the above methods (MANOVA/ANOVA/DIME) along with DBChIP in the multi-sample-multi-factor scenario.

To obtain the correlated negative binomial (NB) data, we first generated correlated MN data in a similar way to the simulation performed in the main paper (Methods). The only difference is that instead of using fixed values comparable to the real data as the parameters to generate the MN data, here we estimated the parameters (mean, variance and correlation) from the real data. The parameters used for generating the "strong", "medium" and "weak" binding signals are based on the means of the top 1%, 5% and 60% of the real data and their values are around 7, 5, 3 while the range of real data is from -3 to 13. As for the background non-binding regions, we used the remaining data other than the top 10% to estimate the parameters and the background mean is around 0.

To transform the MN data to negative binomials, we first used the background mean/variance as the parameters to obtain the p-values of the MN data and used those p-values along with chosen dispersion and probability parameter (r, p) = (0.5, 0.05) to generate the NB data. Since DBChIP was designed for count data, we used NB data to run DBChIP and used log2-transformed NB data to run other methods. While none of DIME/ANOVA/DBChIP was designed for detecting differential binding in multi-sample-multi-factor scenario, we obtained their output statistics from each pair of simulated data tracks. As DBChIP used a common dispersion for all sites as default, instead of the position-specific variance used in the main paper, here we used the average of our estimated position-specific variance from LOESS as the common variance for all binding sites. We then combined their output statistics by Fisher's method to compare with to ours p-values using ROC curve.

In Figure S1, we showed that using NB data, dCaP/DIME/DBCHIP consistently outperformed MANOVA/ANOVA at all levels of binding strengths. While dCaP/DIME/DBChIP performed equally well at strong/medium binding sites, at weak binding seits, dCaP outperformed the others.

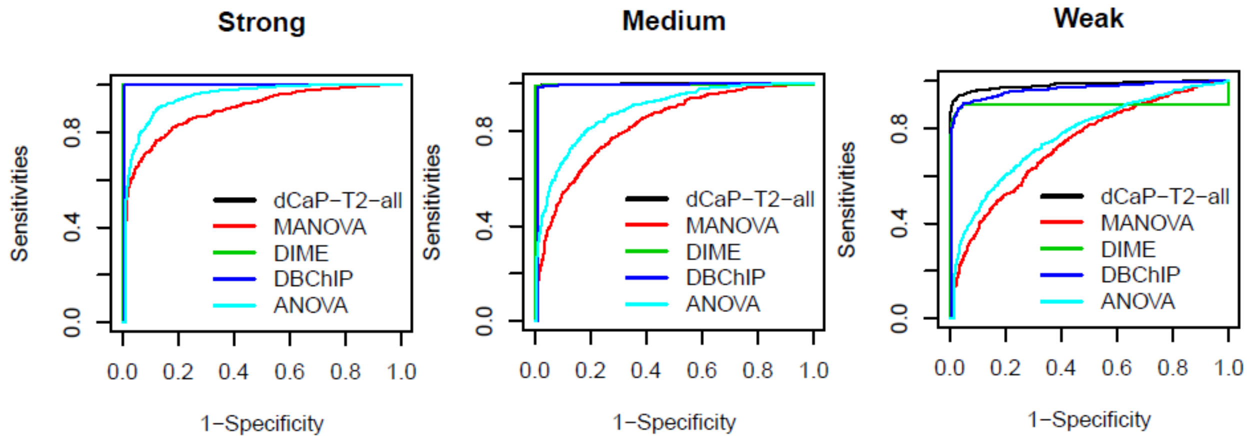

Figure S1. The ROC curves generated using simulated NB data at different levels of binding strengths (strong, medium, weak).
